# Sal-Site: Integrating new and existing ambystomatid salamander research and informational resources

**DOI:** 10.1186/1471-2164-6-181

**Published:** 2005-12-16

**Authors:** Jeramiah J Smith, Srikrishna Putta, John A Walker, D Kevin Kump, Amy K Samuels, James R Monaghan, David W Weisrock, Chuck Staben, S Randal Voss

**Affiliations:** 1 Department of Biology & Spinal Cord and Brain Injury Research Center, University of Kentucky, Lexington, KY, USA 40506

## Abstract

Salamanders of the genus *Ambystoma *are a unique model organism system because they enable natural history and biomedical research in the laboratory or field. We developed Sal-Site to integrate new and existing ambystomatid salamander research resources in support of this model system. Sal-Site hosts six important resources: 1) Salamander Genome Project: an information-based web-site describing progress in genome resource development, 2) *Ambystoma *EST Database: a database of manually edited and analyzed contigs assembled from ESTs that were collected from *A. tigrinum tigrinum *and *A. mexicanum*, 3) *Ambystoma *Gene Collection: a database containing full-length protein-coding sequences, 4) *Ambystoma *Map and Marker Collection: an image and database resource that shows the location of mapped markers on linkage groups, provides information about markers, and provides integrating links to *Ambystoma *EST Database and *Ambystoma *Gene Collection databases, 5) *Ambystoma *Genetic Stock Center: a website and collection of databases that describe an NSF funded salamander rearing facility that generates and distributes biological materials to researchers and educators throughout the world, and 6) *Ambystoma *Research Coordination Network: a web-site detailing current research projects and activities involving an international group of researchers. Sal-Site is accessible at .

## Background

Salamanders of the genus *Ambystoma *are important model organisms in biological research. Their seminal role in experimental embryology and broad utility in laboratory-based science is well known [1]. Ambystomatid salamanders are currently used in multiple areas including olfaction, vision, cardiogenesis, embryogenesis, sensory system development, genomics, and post-embryonic development, including organ and tissue regeneration [2-10]. Moreover, *Ambystoma *is very different from typical laboratory models because much is also known about their ecology, evolution, and natural history. The group is a model in studies of life history and natural phenotypic variation, infectious disease, evolutionary developmental biology, and conservation biology [11-18]. In these respects, *Ambystoma *is a complete model organism system that offers integrative research opportunities spanning the continuum of biological organization.

Recent and on-going molecular resource development is providing new tools for research using ambystomatid salamanders. The Salamander Genome Project (SGP) has recently developed and annotated thousands of expressed sequence tags (ESTs) for *A. mexicanum *and *A. t. tigrinum *[19,20], generated complete mtDNA sequence for 5 different ambystomatid species [21], and completed the first comprehensive genetic linkage map [22]. Markers that have been developed from these ESTs are providing new probes for molecular studies as well as markers for population and quantitative genetics, and phylogenetics [11,15,23]. This recent flurry of resource development stands to greatly increase the utility of ambystomatid salamanders, however there is a need to refine and integrate new resources with existing databases and information. To meet this need, we created Sal-Site [24], a web-portal that functions to integrate new and existing ambystomatid resources. The collation of resources through Sal-Site will enhance communication across the *Ambystoma *community and provide a translational mechanism for researchers working in other model organism systems. Below we describe six resources that are accessible through Sal-Site.

## Salamander Genome Project (SGP)

The SGP [25], supported by the National Center for Research Resources at the National Institute of Health, is currently developing Expressed Sequence Tags (ESTs) and a genetic linkage map (see below). Expressed sequence tags are multifunctional resources because they can be developed for a number of uses, including population and quantitative genetics, comparative genomics, *in situ *hybridization, and functional genomics [21-23]. ESTs are especially useful in the ambystomatid system because sequence information from *A. mexicanum *and *A. t. tigrinum *can be easily extended to enable research in other species [11,15,21], as well as in distantly related vertebrates [24]. Sequences deriving from assembled ESTs are also providing the majority of markers for the *Ambystoma *genetic linkage map [22]. The SGP website was originally developed as a web-interface to allow registered members access to EST and gene mapping data as it was collected. These separate functions are now accomplished through separate but integrated databases that are described below, and there is no longer a requirement for users to register to access these databases. The SGP website now primarily functions to provide information about the project and update progress made in developing genome resources.

## *Ambystoma *EST database (AESTdb)

Although all of the EST sequences developed under the SGP [19,20] are available to the community through NCBI, these ESTs represent an immense collection of unedited data to sift through and sequencing errors are common. The AESTdb [26] was developed in order to organize *Ambystoma *ESTs into edited model RNA sequences and integrate these sequences with related databases. To create the AESTdb, we first performed separate automated assemblies for all available ESTs that have been generated for the species *A. mexicanum *and *A. t. tigrinum *[see also [20]], including a subset of *A. mexicanum *ESTs that are available as unedited contigs from the Axolotl EST Database [19,27]. Automated assembly methods may efficiently correct sequencing errors when large numbers of ESTs are analyzed because it is possible to efficiently identify sequencing and assembly errors against the backdrop of multiple overlapping sequences. However, the SGP has thus far generated an intermediate number of ESTs (~55,000) and many of the assembled contigs contained one or few EST members. As a result, automated methods for error detection were less efficient at detecting sequencing errors and many incorrect base calls were detected upon visual inspection of assembled trace data.

**Figure 1:**
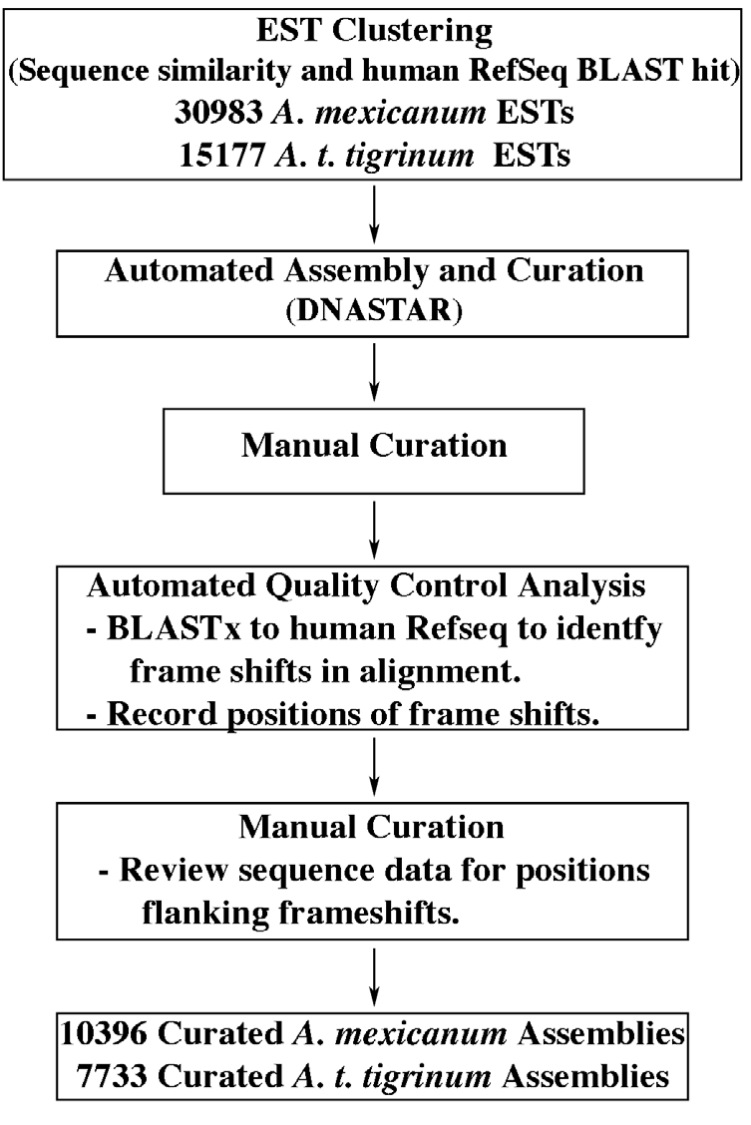
Schematic showing the curation methodology used to assemble and edit *Ambystoma *contigs. The numbers of ESTs and curated assemblies exclude mitochondrial transcripts.

**Figure 2:**
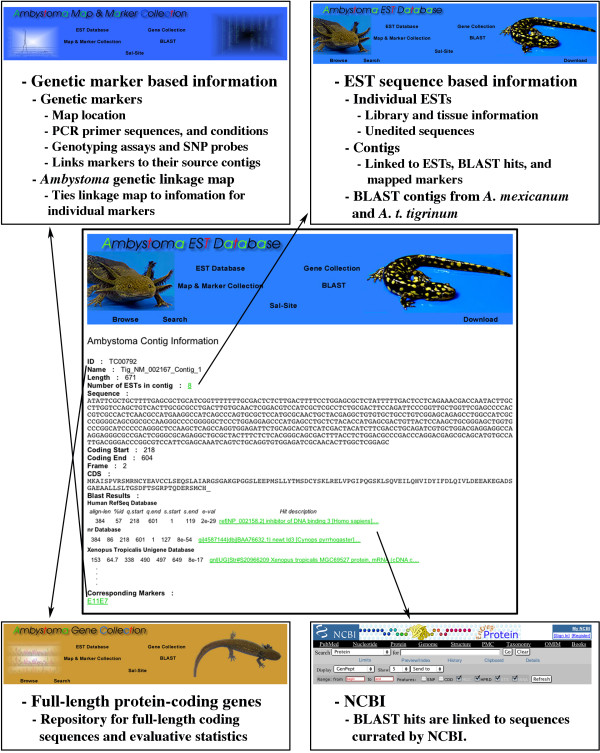
Overview of sequence-based information available through AESTdb and linked resources.

The prevalence of sequence errors among existing *Ambystoma *assemblies necessitated development of a quality-controlled manual editing methodology to minimize error rates within AESTdb contig sequences (Figure 1). All contigs were manually edited in two rounds by visually inspecting aligned trace files, removing low quality sequence ends, and correcting base miscalls. After manual editing, contigs were searched against the human RefSeq and *Xenopus *UniGene databases using the BLASTx and tBLASTx algorithms respectively. If a contig exhibited significant sequence similarity (E value > 1e-7) to a human or *Xenopus *protein-coding sequence, the presumptive ortholog, the reading frame, the translated sequence, and alignment statistics were recorded in individual files that also provide hyperlinks to associate contig sequences with gene and marker files in the *Ambystoma *Gene Collection (AGC) and *Ambystoma *Genetic Map and Marker Collection (AMAP) (Figure 2). For example, if the sequence of a contig was used to develop a PCR amplifiable molecular marker, a hyperlink was included to link the contig file to the molecular marker file in the AMAP database (see below). The AESTdb has a user-friendly web-interface that allows BLAST or textual searching of contigs and access to contig and associated raw EST sequence data.

## *Ambystoma *Gene Collection (AGC)

Analyses of gene function are greatly facilitated by knowledge of full-length RNA and amino acid sequences. The AGC [28] acts as a repository for presumptive full-length sequences from multiple sources, including: AESTdb, sequences parsed from existing databases (e.g. NCBI), and sequences derived from the community at large. We have identified several putative full-length sequences for *A. mexicanum *(n = 940) and *A. t. tigrinum *(n = 717) contigs using the program MuSeqBox [29]. Comparisons against curated full-length protein-coding sequences from the human RefSeq database were performed using three length thresholds for variable amino-terminal and carboxy-terminal regions (20, 50, and 100 amino acids). The majority of putative full-length sequences that were identified showed greater than 50% amino acid sequence identity and greater than 80% sequence coverage when compared to their best human BLASTx hit (Tables 1 and 2). Individual AGC files contain source RNA sequences and predicted amino acid sequences, as well as supplementary fields that provide the best human RefSeq BLAST hit used for analysis of sequence coverage, percent sequence coverage of the best BLAST hit, and percent sequence identity between human and *Ambystoma *amino acid sequences. Files provided in the AGC are hyperlinked to corresponding ESTs and marker sequences in the AESTdb and AMAP as well as NCBI sequence records for corresponding human RefSeq proteins.

**Table 1:** Full-length sequences identified among *A. mexicanum *assemblies

		% Identity				
		0–25	25–50	50–75	75–100	Total
	90–100	1	44	228	241	514
% Coverage	80–90	3	36	101	80	220
	70–80	0	35	78	93	206
	Total	4	115	407	414	940

Distribution of percent amino acid sequence identity between *A. mexicanum *contigs and human RefSeq sequences (% Identity) and percent sequence coverage of corresponding human RefSeq sequences by aligning *A. mexicanum *sequence (% Coverage) for all putative full-length protein coding genes that were identified among *A. mexicanum *contigs.

**Table 2:** Full-length sequences identified among *A. t. tigrinum *assemblies

		% Identity				
		0–25	25–50	50–75	75–100	Total
	90–100	0	37	122	180	339
% Coverage	80–90	0	35	72	64	171
	70–80	2	55	77	73	207
	Total	2	127	271	317	717

Distribution of percent amino acid sequence identity between *A. t. tigrinum *contigs and human RefSeq sequences (% Identity) and percent sequence coverage of corresponding human RefSeq sequences by aligning *A. t. tigrinum *sequence (% Coverage) for all putative full-length protein coding genes that were identified among *A. t. tigrinum *contigs.

## *Ambystoma *Genetic Map & Marker Collection (AMAP)

Although the genomes of ambystomatid salamanders are approximately 10× larger than the human genome [30], the first complete genetic linkage map for any amphibian (including *Xenopus*) was recently assembled using an interspecific mapping cross between *A. mexicanum *and *A. t. tigrinum*. This resource is allowing the mapping of QTL and mutant phenotypes, and the identification of conserved vertebrate syntenies [22,31]. The AMAP website [32] provides images showing the location of mapped markers on linkage groups that correspond to the 14 chromosome pairs in *Ambystoma*. Individual linkage groups can also be accessed as separate datasets in tabular format. These datasets provide precise map distances for all EST and gene-based markers, as well as hyperlinks to separate marker files that provide additional marker specific information including assembly source sequence (hyperlinked to AESTdb records), primer, and polymorphism detection information.

## *Ambystoma *Genetic Stock Center (AGSC)

Recognizing the importance of *A. mexicanum *as a research model, the National Science Foundation has funded continually since 1969 a genetically homogenous collection of animals from which biological materials are distributed throughout the world. This collection is currently housed within the AGSC at the University of Kentucky (formerly the Indiana University Axolotl Colony). The AGSC website [33] provides a user-friendly interface to purchase *A. mexicanum *biomaterials, including embryos, larvae, juveniles, adults, and soon, transgenics. This site also provides a broad range of information including animal care and handling protocols, a history of the axolotl (*A. mexicanum*) collection, descriptions of strains and mutants, staging series for embryos and limb development, and a collection of detailed techniques and protocols. The AGSC periodically distributes an electronic newsletter, called Axolotl Newsletter that contains new developments in *Ambystoma *research and husbandry as well as other items of general interest to the *Ambystoma *community.

## *Ambystoma *Research Coordination Network (ARCN)

The *Ambystoma *Research Coordination Network (ARCN) [34] is comprised of an international group of investigators from diverse organizations. One of the goals is to help participants become better aware of available and emerging resources, biological information, and collaborative opportunities. The ARCN website integrates, via a user-friendly interface, resources from multiple sites, including research profiles and websites of faculty at other institutions, and collaborative research projects.

## System implementation

Sal-Site is implemented using a number of open-source software packages including Apache web server, Perl, CGI, BioPerl, PHP and MySQL. Sal-Site is hosted on a SMP (symmetric multi-processor) PC equipped with two processors, 4GB of RAM and running Linux 2.4.x. We use MySQL 4.0 as the backend Relational Database Management System to store and manage all the information in a robust and efficient way.

## Future directions

Sal-Site is expected to evolve quickly over the next few years as new research and informational resources are developed for ambystomatid salamanders. New methodologies have been developed recently to better enable the system, including the creation of the first transgenic *A. mexicanum *(E. Tanaka, personal communication), techniques to alter gene function *in vivo *[8], and the construction of an Affymetrix GeneChip (Voss, unpublished data). Sal-Site will provide databases and informational resources in support of these and other emerging resources to foster community efforts and make the *Ambystoma *system more accessible to researchers working in other model systems.

## Authors' contributions

JJS participated in sequence curation, development of manual curation methods, database development, and participated in drafting the manuscript. SP participated in sequence curation, development of manual curation methods, database development, system implementation, and participated in drafting the manuscript. JAW, DKK, AKS, JRM, DWW, CS participated in sequence curation and development of manual curation methods. SRV conceived of the study and participated in drafting the manuscript. All authors have read and approved the final manuscript.
